# Current Hypotheses on How Microsatellite Instability Leads to Enhanced Survival of Lynch Syndrome Patients

**DOI:** 10.1155/2010/170432

**Published:** 2010-06-10

**Authors:** Kristen M. Drescher, Poonam Sharma, Henry T. Lynch

**Affiliations:** ^1^Department of Medical Microbiology and Immunology, Creighton University School of Medicine, Omaha, NE 68178, USA; ^2^Department of Pathology, Creighton University School of Medicine, Omaha, NE 68178, USA; ^3^Department of Preventative Medicine, Creighton University School of Medicine, Omaha, NE 68178, USA

## Abstract

High levels of microsatellite instability (MSI-high) are a cardinal feature of colorectal tumors from patients with Lynch Syndrome. Other key characteristics of Lynch Syndrome are that these patients experience fewer metastases and have enhanced survival when compared to patients diagnosed with microsatellite stable (MSS) colorectal cancer. Many of the characteristics associated with Lynch Syndrome including enhanced survival are also observed in patients with sporadic MSI-high colorectal cancer. In this review we will present the current state of knowledge regarding the mechanisms that are utilized by the host to control colorectal cancer in Lynch Syndrome and why these same mechanisms fail in MSS colorectal cancers.

## 1. Introduction

Although Warthin described a classic cancer prone family (cancer family G) in the early 1900's [1], relatively little attention was given to “cancer families” until the 1960s when Lynch described two large Midwestern cancer kindreds and hypothesized that they were due to hereditary factors [2]. This hypothesis was widely dismissed and other theories regarding the origins of cancer, such as exposure to an environmental agent, were favored. Lynch also assessed the cancer status of relatives of Warthin's family G over six generations, revealing an autosomal dominant pattern of inheritance [3]. The hypothesis that cancer risk in such families could be inherited was validated in the 1990s when multiple groups identified regions of the genome associated with colorectal cancer development [4–6]. The recognition that some cancers were hereditary was significant, as it permitted increased surveillance and testing of individuals who were at the greatest risk for disease development. This also allowed patients to exercise some level of control (i.e., seek early diagnosis) over disease outcome. 

Lynch Syndrome is a hereditary colorectal cancer (CRC) syndrome. Patients with Lynch Syndrome experience early-onset CRC as well as an increased risk of developing certain extracolonic cancers [7–17] In sporadic cancers, there are often point mutations in tumor suppressor genes and protooncogenes including K-ras, p53 and APC [18–23]. These mutations are seldom observed in Lynch Syndrome cancers [24–26]. The primary defect in Lynch Syndrome cancers results in increased microsatellite instability due to a mutation in the DNA mismatch repair genes; microsatellite instability is not common in sporadic cancers [4–6, 27]. Lynch Syndrome tumors are more poorly differentiated compared to other CRCs and are frequently characterized by an excess of mucin, a Crohn's-like reaction, and signet-cell features [28]. These features would be expected to indicative of a worse prognosis but represent a paradox in the case of Lynch Syndrome patients. Despite these characteristics associated with a poor prognosis, Lynch Syndrome patients experience enhanced survival compared to patients with sporadic CRC [29]. The survival advantage of these patients is of great interest to scientists and physicians alike and several different hypotheses have been considered in contributing to this. Factors that may be responsible for improved survival in Lynch Syndrome patients include increased infiltration of the tumor with T cells [30–33], reduced viability of tumor cells due to genomic instability [34, 35], and the diploid nature of Lynch Syndrome tumors [36, 37].

The concept that some tumors can be controlled by the immune system is over 50 years old. It was observed that if a tumor was resected from a mouse, reinjection of the same tumor into the same mouse resulted in no tumor growth. Injection of the tumor into a naïve mouse resulted in tumor growth [38]. While these early experiments provided insight into the role of the immune system in controlling cancer, many tumors are weakly immunogenic as they are derived from host tissue. Ergo, the host cannot mount a strong, effective immune response to the tumor. The immune system can be exploited to control tumors that express foreign antigens (such as the case with human papillomavirus and cervical cancer vaccine). Lynch Syndrome tumors have high levels of tumor infiltrating lymphocytes (TILs) [30–33, 39–50], suggesting that tolerance does not exist to some peptides expressed by these tumors. This paper will focus on the potential role that the immune system may play in enhancing prognosis in Lynch Syndrome, although other cellular mechanisms may also influence host well-being. Some of these mechanisms, such as the accumulation of defects in genes associated with tumor viability [34, 35], may work in conjunction with the immune mechanisms described herein.

## 2. Overview of Lynch Syndrome

Lynch Syndrome, sometimes referred to as hereditary nonpolyposis colorectal cancer (HNPCC) comprises 2–5% of all colorectal cancer cases [51–53] and develops due to an autosomal dominant mutation in at least one of the DNA mismatch repair genes [4–6, 51]. As these mutations are highly penetrant, multiple generations of a family are frequently affected by cancer [51, 54]. Defective DNA mismatch repair results in the accumulation of insertions and deletions within short repetitive sequences called microsatellites. Alterations in the length of these microsatellites lead to a condition known as microsatellite instability (MSI). Tumors from individuals with defects in mismatch repair genes, most common of which are mutL homolog 1 (MLH1) and mutS homolog 2 (MSH2), have high levels of microsatellite instability (MSI-high). It is important to note that while not all cancer prone families with MSI-high CRCs are classified as Lynch Syndrome, nevertheless, many of the features of Lynch Syndrome are applicable to MSI-high CRC. MSI-high sporadic CRCs are often caused by inactivation of mismatch repair genes due to promoter hypermethylation of MLH1 [55–58]. 

The designations of Lynch Syndrome and HNPCC have been used interchangeably for many years. However, the term HNPCC may also include cases of CRC that have features associated with Lynch Syndrome but lack a mutation in one of the mismatch repair genes [59]. These cases of CRC are characterized as having Amsterdam criteria positivity but lacking a mismatch repair germline mutation and are referred to as familial CRC type X [60]. While in this paper we have focused on Lynch Syndrome patients, we will use the term HNPCC when that term was used in the papers being discussed. Some studies have shown that patients with MSI-high non-Lynch Syndrome CRC have a survival advantage over patients microsatellite stable CRC [29], although this remains controversial in that recent studies have failed to confirm this finding [61].

The clinical and pathology features associated with Lynch Syndrome are distinct from those of sporadic CRC [62, 63]. Tumors from Lynch Syndrome patients are typically located proximal to the splenic flexure [4, 64] and develop at an earlier age (<50 years old) than colorectal tumors from sporadic cases of cancer [62, 65]. Lynch Syndrome tumors are also less likely to metastasize than non-Lynch Syndrome tumors despite the presence of multiple colorectal tumors [41, 64, 65]. Patients with Lynch Syndrome are also predisposed to developing metachronous colorectal cancers and extracolonic cancers including cancers of the small bowel, ureter, renal pelvis, pancreas, biliary tract, endometrium, ovaries, and brain at a higher than expected rate [7, 9, 10, 12–15, 66, 67]. An increased risk of breast and prostate cancer development is not usually associated with Lynch Syndrome, although some studies have suggested otherwise [16, 17, 68]. This topic has been extensively reviewed recently [69]. 

While several groups have studied the cumulative lifetime risk of Lynch Syndrome patients developing extracolonic cancers [8, 66, 70–75], data may be skewed due to limited sample sizes or overrepresentation of specific mutations in the mismatch repair genes. A recent study examined the risk of developing extracolonic cancers in 121 Lynch Syndrome families in the United Kingdom [76]. Mutations in MLH1 were present in 51 families, while 59 families had mutations in MSH2, and 11 families had mutations in MSH6 [76]. Eight hundred thirty nine mutation carriers were analyzed and 282 extracolonic cancers identified [76]. Females had a significantly higher cumulative lifetime cancer incidence compared with males, consistent with the association of gynecological cancers (ovarian and endometrial) with Lynch Syndrome. The risk of endometrial cancer development in women with MSH6 mutations was approximately twice the risk in women with MLH1 or MSH2 mutations [76]. Males had an increased risk of gastric cancer compared to females in this study, although upon further analysis of these data there was minimal risk for gastric cancer development in individuals born after 1935. Individuals with MSH2 mutations had a higher cumulative lifetime risk of developing primary CNS tumors compared to nonmutation carriers [76]. 

The name, hereditary nonpolyposis colorectal cancer, may be somewhat misleading as adenomas that subsequently develop into tumors are present in Lynch Syndrome patients [77–80]. The presence of more than 10–15 adenomas is extremely unusual in Lynch syndrome and may suggest attenuated familial adenomatous polyposis [81, 82]. Analysis of adenomas from Lynch Syndrome patients demonstrates that a high percentage of the adenomas (between 60 and 90%) are deficient in mismatch repair proteins, indicating that the adenomas have similar phenotypes as the colorectal cancers [79, 80, 83–88]. Data from clinical surveys of colorectal cancer patients suggest that adenomas from Lynch Syndrome patients may be more aggressive than sporadic adenomas [89–94]. The mean time from adenoma to carcinoma formation is 2-3 years in Lynch Syndrome patients and 6–8 years in sporadic colorectal cancer. The adenomas formed in Lynch Syndrome may be more difficult to detect due to the flat morphology that can be observed in some Lynch Syndrome patients [95–98].

The Amsterdam criteria were developed in 1991 to assist in the diagnosis of Lynch Syndrome [62]. Individuals who meet the Amsterdam criteria have a better prognosis than patients with microsatellite stable colorectal cancer [11, 41, 99, 100]. A study by Buckowitz et al. estimated the five-year survival rate for MSH-high CRC that met the Amsterdam criteria at 88% versus 56% for microsatellite stable CRC [41]. After adjusting for age and disease stage at time of diagnosis, Watson et al. also demonstrated a survival advantage for Lynch Syndrome CRC patients compared to patients with sporadic CRC [11]. Of particular interest to both physicians and basic scientists is why patients with Lynch Syndrome enjoy this survival advantage over patients with sporadic CRC. This finding, in combination with the increased rate of primary extracolonic cancers [7, 9, 10, 12–15, 66, 67] and decreased rates of metastases [41, 64, 65], suggests that the Lynch Syndrome patients develop significant defenses to the cancer. A better understanding of what features of Lynch Syndrome are key to the observed enhanced survival would likely provide insights into not only the predominant mechanisms employed by the host defense system to control CRC, but also could permit the development of more effective therapies for both Lynch Syndrome and sporadic CRC patients. 

## 3. Increased lymphocyte Infiltration of Tumors in Lynch Syndrome Patients

**Figure 1:**
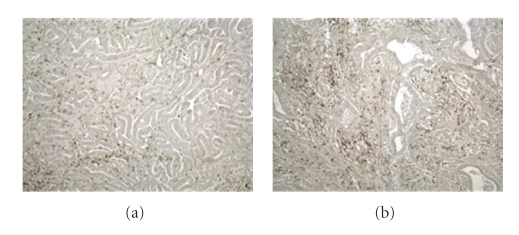
Tumors from Lynch Syndrome patients experience increased infiltration of tumors with CD3+ lymphocytes (b) compared to non-Lynch Syndrome patients (a).

The potential positive impact on disease outcome that dense infiltration of lymphocytes in various tumors has been a subject of considerable debate and speculation [30–33, 39–50]. A recent study of CRCs that did not take into consideration the MSI status of the tumor found that patients with tumors infiltrated with increased levels of cytotoxic and memory (CD45RO+) T cells had improved outcome as compared with patients with low levels of cytotoxic and memory T cells [50]. This particular study focused on patients with stage I and II CRC. Several studies have examined MSI-high colorectal carcinomas and characterized both the number and phenotype of the tumor infiltrating cells [30, 31, 41, 43, 47–49, 101, 102]. Studies by Smyrk et al. described an increased level of tumor infiltrating lymphocytes (TILs) in CRCs with high levels of microsatellite instability. In this study, TIL's in MSS, MSI-low and MSI-high CRCs were correlated with microsatellite status, and it was determined that tumors from HNPCC and other MSI-high CRCs had increased lymphocyte infiltration compared to MSI-low or MSS tumors [47] (Figure 1).

The prognostic significance of the presence or absence of TILs is a function of both the specific form of cancer, as well as the nature of the infiltrate. In one study, nine of 11 MSI-high patients with high levels of CD8+ intratumoral lymphocytes experienced tumor-free survival [48], suggesting a correlation between CD8+ T cell infiltration of a tumor and a positive disease outcome. The levels of activated cytotoxic intraepithelial lymphocytes were significantly increased in MSI-high CRCs as compared to MSS CRCs [30, 31, 48]. Furthermore, lymphocytes from MSI-high CRC are more likely to express high levels of CD8, a marker of cytotoxic T cells, as well as granzyme B and perforin than non-MSI-high tumors [30]. Together these data support a role for the CD8+ T cells in the control of Lynch Syndrome tumors by a cytotoxic mechanism aimed at killing tumor cells. Only a small percentage of CD56+ cells (natural killer cells; NK cells) was found, indicating that the majority of infiltrating lymphocytes were likely CD8+ cytotoxic T cells [30]. These data are consistent with immune activation of the adaptive immune system. 

CRCs that arise through mechanisms other than impairment of the mismatch repair genes (for example, mutations in k-ras or p53) also have some additional characteristics that provide clues to the reduced survival times of individuals with non-MSI-high cancers. In one study, tumors from MSS and non-HNPCC MSI-high patients were examined using quantitative RT-PCR. This particular study also compared alterations between tumor and nontumor tissue. MSS tumors had enhanced levels of Foxp3, IL-6, IL-17, and TGF-*β* transcripts as compared to MSI-high tumors [42]. Increased levels of Foxp3, a marker of regulatory T cells (T regs), may have significant impact on the ability of the host to promote a sufficient immune response, as T regs downregulate inflammation in an antigen-specific manner. The alterations in cytokines would suggest that an environment conducive to tumor growth and angiogenesis is present in the MSS tumors [103, 104], creating a milieu that would favor the development of metastases. In another study that did not consider the microsatellite status of the tumor, increased numbers of T regs were observed in patients with CRC [105]. Interestingly, increased numbers of Tregs correlated with more limited disease versus metastatic disease. Given that the majority of CRCs are MSS, it is likely that these tumors are overrepresented in this study [105]. A recent study from our group found no differences in the level of T reg infiltration of MSS and MSI-high CRC using CD25 as a marker of T regs [31]. 

The available data suggest that (1) Lynch Syndrome patients have increased CD8+ cytotoxic T cells within the tumor; (2) patients with sporadic MSS CRC have increased levels of transcripts within the tumor that would promote tumor growth and metastases. Adenomas from HNPCC patients are also more likely to have adenoma infiltrating lymphocytes (AILs) compared to adenomas from control patients [92, 106], indicating that the processes responsible for recruitment of the lymphocytes to the tumor are established early in the development of the tumor. What would account for the increased number of TILs in Lynch Syndrome compared with sporadic CRC cases? Are these TILs the key to enhanced survival? What are the specificities of the T cell receptors expressed by the TILs? The answers to these pertinent questions likely lie in the fundamental defect in Lynch Syndrome patients' DNA mismatch repair genes. The role of the defective DNA mismatch repair genes in generating targets for immune response is discussed below.

## 4. How Could Defects in the DNA Mismatch Repair System Provide a Survival Advantage to HNPCC Patients?

### 4.1. Overview of the DNA Mismatch Repair System

Defects in the DNA mismatch repair system increase the error rate of replication by 100- to 1000-fold [107–109]. Areas of the genome that contain repetitive sequences (microsatellites) are particularly susceptible to insertions and deletions of bases during the replication process [4, 110]. Strand slippage of DNA polymerase and inefficient proofreading contribute to the vulnerability of microsatellites to errors during DNA replication. The mismatch repair proteins are mediators for fixing these errors, thus allowing the host to maintain genomic fidelity [111–118]. To repair errors that occur during DNA replication, the mechanism of repair must (a) recognize the error; (b) remove the incorrect bases; (c) resynthesize the DNA. Several excellent reviews are available on the mismatch repair system [119–122]. In humans, the error in the DNA sequence is recognized by a heterodimer consisting of MSH2 and either MSH3 or MSH6. This heterodimer binds the double stranded DNA at the site of the error, which then permits binding of a second complex consisting of MLH1 and either postmeiotic segregation increased 2 (PMS2), PMS3, or MLH3 in an ATP-dependent manner. The binding of the second heterodimer results in the movement of the complex along the DNA until it encounters the PCNA:DNA polymerase and displaces it from the DNA. Exonuclease I then excises nucleotides from the site of the DNA polymerase to the site of the error on the daughter strand thereby permitting resynthesis of the daughter strand. The daughter strand is then resynthesized. Because MSH2 and MLH1 do not have alternative “stand in” proteins like their binding partners, germline mutations in these genes are most commonly associated with defects in the DNA mismatch repair system and increased levels of microsatellite instability in Lynch Syndrome. MSH6 and PMS2 mutations are less common [111, 114, 123–127]. Regardless of which mismatch repair gene is mutated, members of a given Lynch Syndrome family normally have the same mutation, although the first occurrence of cancer may differ in terms of location and age of onset.

### 4.2. Consequences of Defects in DNA Mismatch Repair Genes

During T cell development, immature T cells enter the thymus and undergo positive and negative selection. As a result of completing this process successfully, the host's T cell compartment contains a repertoire of cells that are self-restricted and self-tolerant [128–130]. Under most conditions the T cells will recognize peptides bound to the host's MHC molecules and will not have T cells reactive to peptides that are derived from self proteins. Failure to achieve the elimination of the self-reactive T cells can result in autoimmune diseases such as type 1 diabetes and rheumatoid arthritis. Because tumors are derived from host tissue, the immune system often does not have T cells in the repertoire that will react sufficiently to the tumor. Tumors that are immunogenic typically express mutated or aberrantly expressed proteins. The mismatch repair system is one mechanism to generate mutated proteins, and the key to generating these proteins are mononucleotide repeats that are found with in the genome.

Over 30 human genes containing mononucleotide repeats of greater than 7 bases have been identified [122]. Common repetitive sequences in the human genome are (A)n/(T)n and (CA)n/(GT)n [119, 131]. These genes are more vulnerable to mutations than genes that do not contain repetitive sequences. These repetitive sequences are found in a number of coding regions of genes (coding microsatellites) that are involved in apoptosis (APAP-1, BAX, BCL-10, Caspase-5, FAS, RIZ) as well as mismatch repair genes (MLH3, MSH3, MSH6). Growth factors and their receptors (ACTRII, GRB-14, IGFIIR, TGF*β*RII, and WISP3) are also affected by the loss of mismatch repair functions [122, 132]. Because there are insertions and deletions in the repetitive sequences within the coding region of these genes, tumors from Lynch Syndrome patients often have alterations in the translational reading frame of the affected gene [132, 133]. This can result in the generation of altered proteins unique to the tumor [134]. MSI-high tumors are unique in that a frameshift mutation in the translational reading frame of the gene can result in the generation of new peptides, some of which may be immunogenic and recognized by the host's immune system as foreign. Because these frameshift peptides (FSPs) are unique to the tumor and not present in other areas of the patient's body, it is unlikely that central tolerance to these FSPs has developed, and T cells with reactivity to these peptides may exist in the host. Because of this, FSPs have the potential to serve as a tumor-specific target for the immune system in cancers (such as Lynch Syndrome) that have high levels of MSI. T cells, particularly CD8+ T cells, could target these FSPs, resulting in cytotoxic killing of tumor cells. 

As early as 2001, the potential for FSPs to interact with and stimulate T cell mediated immune responses was considered [134, 135]. Modeling immune responses in vitro, Linnebacher et al. demonstrated the ability of a peptide generated from a frameshift of the TGF*β*IIR gene to stimulate an in vitro immune response as measured by IFN*γ* production and target cell lysis [134]. In a similar study, Sæterdal et al. identified frameshift peptides derived from TGF*β*RII and BAX were capable of stimulating T cell mediated immune responses [136]. In this study, two of three MSI-high and three of three HNPCC patients had immune responses to the TGF*β*RII-derived FSP. Together, these two studies suggest that the requirements that are needed to generate a sustained immune response to at least some FSPs are present in some MSI-high patients. The ability to detect responses in both the CD4+ and CD8+ T cell compartments is significant in that CD4+ T cells are normally required to generate strong, sustainable CTL responses. 

While FSPs have been determined to be immunogenic [134, 136], a more recent study by Schwitalle et al. has further explored the biological relevance of these FSPs in vivo in HNPCC CRC patients and healthy HNPCC mutation carriers [137]. TILs were isolated from tumor tissue of MSI-H colorectal cancer tumors and assayed for cytotoxic potential. TILs from HNPCC tumors were reactive to MSI-high but not MSS tumor cells, indicating that TILs were specific for peptides expressed by the MSI-high tumors. TILs were examined for reactivity to FSPs as measured by IFN*γ* release following exposure of the T cells to 24 FSPs that were derived from 14 genes. Cytotoxic T cells were identified that were reactive to FSPs including those derived from TGF*β*RII, caspase 5, OCT, and AIM-2 [137]. The implication of these studies is that individuals with MSI-high tumors have (1) tumors that are immunogenic; and (2) immune cells capable of focusing a targeted attack on the tumor. Reactivity to FSPs was also detected in healthy HNPCC carriers, suggesting that either (1) a protective response occurred in individuals that did not develop cancer; or (2) undetected adenoma development had occurred in some patients, and the immune response was already activated. 

If the FSPs that are found in MSI-high are immunogenic, then why cannot the immune system rid the body of the tumor? Data demonstrate that immune evasion strategies are likely invoked by the tumor. Because antigen presentation to CD8+ T cells requires the formation of a peptide-MHC Class I complex several groups have studied the expression of class I on the surface of colorectal cancer tumors. There are several mechanisms that could result in loss of MHC class I expression on a cell's surface. Most commonly, these defects are related to the loss or mutation of *β*2 microglobulin, or defects in the antigen processing components (for example, LMP components), or defects in proteins involved in peptide loading (TAP1/TAP2, tapasin) [138–142]. 

In considering that Lynch Syndrome patients have increased levels of CD8+ T cells within the tumors, as well as fewer metastases, it would be logical to hypothesize that within the tumor environment there is a robust immune response occurring. From the perspective of the tumor, strong selective pressure would be expected to promote the outgrowth of Class I negative cells, thereby permitting these tumor cells to evade the MHC class I mediated immune response. In examining MSS and MSI-high CRCs, distinct mechanisms of class I loss were identified [139, 140, 143]. Several recent studies have focused on how the presence of the CD8+ T cells could control antitumor responses and what the potential targets of the CD8+ T cells are. Focused answers to these questions require an explanation as to why the proposed mechanism are effective in Lynch Syndrome patients, but not microsatellite stable colorectal cancer patients.

The concept that *β*2 microglobulin loss occurs in colorectal cancer tumors with high levels of microsatellite instability was examined by Bicknell et al. [139]. In this study, the frequency of *β*2 microglobulin mutations was determined in various cancers inclusive of colorectal, melanoma, breast, ovary, and lymphoma. Mismatch repair mutations were more likely to be associated with *β*2 microglobulin mutations than other defects in the antigen processing machinery [139]. Furthermore, tumors that were not associated with defective mismatch repair genes were not likely to have *β*2 microglobulin mutations.

In a study by Kloor et al., a high rate of total HLA class I loss was observed in MSI-high tumors (~60%) compared to MSS tumors (~30% loss) [94]. Further molecular analysis of the tumors revealed mutations in *β*2 microglobulin (~30% of the tumors with Class I loss) and defects in TAP1 or TAP2 (17% of the tumors with class I loss). The loss of *β*2 microglobulin expression was likely due to frameshift mutations resulting from the high level of microsatellite instability [139].

A more recent study by Dierssen et al. [143] utilized CRCs that were location matched (that is, both sporadic MSI-high and LS tumors were right sided) [105]. In this study, LS tumors were found to be significantly associated with a mutation in *β*2 microglobulin, while sporadic MSI-high tumors were more likely to be the result of a mutation in the antigen processing machinery such as the proteosome (LMP2, LMP7, LMP10, and MBI), TAP1, TAP2, or chaperone proteins (calnexin, calreticulin, ERP57, and tapasin).

While both MSI-high right-sided tumors and HNPCC had increased loss of class I expression on their surface, class I expression was intact in MSS tumors. The basis of class I loss was further investigated, and *β*2 microglobulin mutations were correlated with class I loss in HNPCC cases. In MSI-high sporadic right sided tumor cases, multiple components of the antigen processing machinery were found in all but 30% of the cases. TAP1/2, tapasin and LMP2 mutations were the most common defects. MSS tumors appear to become HLA negative via a third pathway—loss of heterozygosity at chromosome 6p21.3, demonstrating that the evolution of these three forms of colorectal cancer are unique in the host [105]. Furthermore, these underlying differences suggest that distinct treatment options are likely required. Indeed, microsatellite instability may influence the response to certain treatments, in particular 5-fluorouracil (5-FU) adjuvant therapy in patients with MSI-high CRCs. Ribic et al. [144] demonstrated that patients with MSI-high stage II or stage III CRCs did not benefit from adjuvant chemotherapy compared to patients with stage II or stage III MSS or MSI-low CRC. 

In examining the presence or absence of HLA class I expression in MSI-high cancers in the context of the tumor stage, and the presence or absence of distant metastases, intuitively one may suggest that the loss of *β*2 microglobulin and/or class I expression would be increased in tumors that have metastasized. The rationale behind this hypothesis would be that in the absence of class I expression TILs cannot appropriately perform their immunosurveillance function and the tumor would metastasize from its primary site.

The association of *β*2 microglobulin mutations on CRC progression was examined. In this study, tumors were stratified by stage [138]. Of the MSI-high adenomas examined, almost 16% were positive for mutations in *β*2 microglobulin, indicating that these mutations are apparent prior to tumor development. *β*2 microglobulin mutations accumulated as tumors progressed from grades 1 (26.7% positive) through grade 3 (43.5% positive). Of great interest however, was that no stage 4 cancers in Lynch Syndrome had *β*2 microglobulin mutations, suggesting that *β*2 microglobulin may be involved in the metastatic process [138]. This surprising finding suggests mechanisms other than class I-mediated cytotoxic processes are involved in tumor control. 

If, after a period of time, increased loss of *β*2 microglobulin is found in colorectal cancers with high levels of microsatellite instability, what mechanisms may be involved in tumor control? Natural killer (NK) cell-mediated killing has, as a requirement, downregulated levels of MHC class I on the surface of the targeted cell. In this scenario, loss of *β*2 microglobulin would result in loss of Class I, leading to targeting of the tumor cell by NK cells. A small number of studies have suggested that this mechanism of killing is plausible in MSI-high colorectal cancers. Studies by Menon et al. determined that tumors that were MLH-1 negative and had decreased levels of HLA were more likely to contain CD57+ cells [49]. Lack of class I expression has also been associated with NK cell regulation and activation. Studies by Dierssen et al. [143] have shown that CRCs from HNPCC patients have decreased expression of HLA class I on their surface [105]. 

A study examining the association between the number of myeloperoxidase positive cells and microsatellite status was performed by Roncucci et al. [145]. Myeloperoxidase is a lysosomal enzyme highly expressed by neutrophils, and to a lesser degree, by macrophages and monocytes. This marker was used to assess the level of colonic inflammation, as it is a key component of the neutrophil cytotoxic granules. This study found a strong correlation between myeloperoxidase staining and the presence of abnormal crypts, which are presumed precursors of adenomas. Once tumors had formed, increasing levels of myeloperoxidase staining was observed in both MSS and MSI-high colorectal cancers. However, MSI-high tumors had significantly higher levels of myeloperoxidase immunoreactivity as compared with MSS tumors. The increase in myeloperoxidase staining in MSI-high versus MSS tumors is consistent with an increased inflammatory response in the MSI-high tumors [145]. This study also suggests that mediators of the inflammatory response (in particular neutrophil products) may drive tumor development.

The balance between cell proliferation and cell death is known to influence tumor progression and development. It is key to consider not only the number of cells undergoing apoptosis but also the ratio of cells experiencing apoptosis to the number of cells undergoing proliferation in the context of tumorigenesis. Examination of these parameters is MSI-high and MSS/MSI-low colorectal cancers found that MSI-high tumors had significantly decreased levels of proliferation compared to MSI-low or MSS colorectal cancers [146]. 

When considering the role of the immune system in tumor control, one must also consider the inherent characteristics of the tumor, and what potential mechanisms can be used by the tumor to evade immune surveillance in vivo. Some studies have demonstrated that FasL expression on tumor cells can interact with Fas-expressing TILs and trigger apoptosis of the Fas-expressing TIL [32, 45, 147]. This situation has been termed “Fas counterattack.” When Fas counterattack occurs, individuals with increased numbers of TILs had a worse prognosis than individuals with low numbers of TILs. In these studies, high levels of Fas L expression by tumors results in apoptosis of the invading TILs [32, 45, 147].

A recent study by Koornstra et al. examined FasL expression and apoptosis in colorectal tumors from Lynch Syndrome patients to assess whether Fas counterattack had a role in Lynch Syndrome pathology or clinical manifestations, in particular, the accelerated transformation from adenoma to tumor [148]. Enumeration of the number of apoptotic TILs and tumor cells in Lynch Syndrome and sporadic colorectal cancer, as well as the number of FasL positive cells, revealed no association between FasL expression, apoptosis, and the number of TILs in Lynch Syndrome patients. It is unclear as to whether the rate of apoptosis in the adenomas is different from the levels observed in the tumor. A recent study demonstrated reduced levels of apoptosis were observed in HNPCC adenomas [106]. The decrease in apoptosis, which may in part be attributed to frameshift mutations in apoptosis-related genes, may account for the relatively fast transition from adenoma to tumor in HNPCC patients.

## 5. Current Model of Tumor Control in Lynch Syndrome

**Figure 2:**
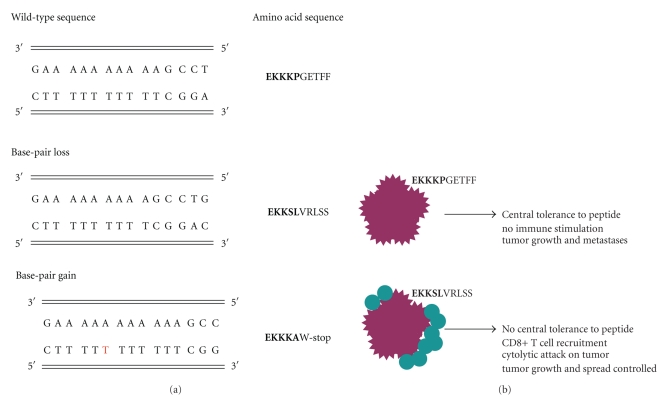
Defects in DNA mismatch repair genes leads to the generation of immunogenic Peptides expressed by the tumor. (a): The first sequence is a partial sequence of the TGF*β*RII gene. Note the ten base stretch of adenines. The area of the gene is susceptible to strand slippage during replication. When DNA mismatch repair genes are mutated, base-pairs can be lost or added during DNA replication. Additions or deletions result in an altered reading frame. Using the TGF*β*RII gene as an example, the amino acid sequence is shown on the right. Bolded animo acids are those amino acids represented by the nucleotide on the left. Unbolded sequences are those that are predicted based on the mutation, using the nucleotide sequence of TGF*β*RII (120). (b): Following the generation of novel frameshift peptides to which the immune system has not developed central tolerance to, the host can generate a robust immune response to the peptides that are uniquely expressed by the tumor.

Based on the current state of knowledge, we propose the following model for the control of tumors in Lynch Syndrome patients (Figure 2). CRCs in Lynch Syndrome patients have a high degree of microsatellite instability. Mutations in the DNA mismatch repair genes results in DNA strand slippage and the generation of novel frameshift peptides within the tumor. Because the host has not generated immune tolerance to these peptides, these neopeptides are highly immunogenic and the host generates a strong inflammatory response. Immune cells are heavily recruited to the tumor site and the infiltrate is dominated by CD8+ T cells. Because of the robust nature of the anti-tumor response, there is selective pressure for the outgrowth of tumors that have mutations in *β*2 microglobulin, which results in reduced antigen presentation at the tumor site. Indeed, mutations in *β*2 microglobulin are more frequent as tumor grade increases. Of interest however, is the lack of these mutations in grade IV metastatic Lynch Syndrome tumors. Tumors in Lynch Syndrome patients do not appear to invoke other immune subversion processes such as Fas counterattack of the infiltrating lymphocytes, as evidenced by work correlating FasL expression with tumor cell apoptosis and the number of TILs [148]. 

The hypothesis that the immune system of a host with a mutation in a tumor suppressor gene (that is, one of the mismatch repair genes) could develop a strategy to overcome this mutation (that is, developing a strong anti-tumor response) is intriguing. The increased survival of Lynch Syndrome patients also brings up some fascinating questions from an evolutionary standpoint: (1) given that Lynch Syndrome patients have a better prognosis than non-Lynch Syndrome patients and the basis for the Syndrome is encoded in the germ-line is pertinent to the question as to whether there will there be increased rates of Lynch Syndrome (relative to non-Lynch Syndrome CRCs) in generations to come? (2) does the enhanced immune response, lack of metastatic events, and enhanced survival lead to increased dissemination of Lynch Syndrome? Unfortunately, one cannot easily assess this question either retrospectively or prospectively. The history of Lynch Syndrome is too short to accurately grasp the rates of this form of CRC over time. 

It is likely that Lynch Syndrome is underreported due to the required testing. Unlike viruses or bacteria which have extremely short generation times, the amount of time it would take to observe such changes in disease incidence in the human population would be beyond our lifetimes. However, banking of genetic material could be helpful in assessing the evolution of CRC in future generations. It is also important to consider that the trend towards replacing microsatellite instability screening with immunohistochemistry [149–152], a less expensive and more widely available diagnostic tool, will also affect our perceptions of the rates of Lynch Syndrome, and thus also must be factored into any estimates of changes in disease incidence over time. 

## 6. Future Directions

Despite the autosomal dominant inherited predisposition to Lynch Syndrome, there is significant variability in whether an individual will develop cancer. With the relative commonplace nature of genome-wide association studies, several single nucleotide polymorphisms have been identified which appear to influence colorectal cancer development. Six variants associated with colorectal cancer development have been identified on five chromosomes—8, 10, 11, 15, and 18. Recently, Hans Vasen's group in The Netherlands performed a large (675 individuals in 127 families) study that involved genotyping the six candidate loci (8q24.21, 8q23.3, 10p14, 11q23.1, 15q13.3, and 18q21.1) in Lynch Syndrome patients and analyzed the association between specific variants and the risk of colorectal cancer development. Two single nucleotide polymorphisms (SNPs) were identified—rs6983266 on chromosome 8q24.21 and rs3802842 on chromosome 11q23.1—which increase the risk of developing CRC in Lynch Syndrome individuals [153]. 

Microarray analysis of MSI-high and MSS cancers has bolstered some of the immunohistochemical findings [154]. Granzyme A and granulysin were found to be upregulated in MSI-H colorectal cancers as compared with MSS cancers, indicating the immune mediators involved in cytotoxic lymphocyte functions are upregulated and the cells in the tumor are likely to be activated. In addition to these mediators, this study also found an increase in the expression of two heat shock proteins—HSP-70 and HSP-110. Heat shock proteins (also called stress proteins) are inducible molecules involved in both the innate and adaptive immune responses, and often act as proinflammatory molecules [154]. 

Further clues into how the host controls tumor growth and metastasis will likely be obtained from the types of studies described above utilizing large-scale screening of gene transcription, as well as high-density molecular array to identify unique characteristics of the genomes of Lynch Syndrome patients. Understanding these factors, regardless of whether they are specific to certain Lynch Syndrome mutations or whether they are reflective of all Lynch Syndrome patients, will provide insights into the mechanisms of protection invoked in these individuals.
